# Central obesity may account for most of the colorectal cancer risk linked to obesity: evidence from the UK Biobank prospective cohort

**DOI:** 10.1038/s41366-024-01680-7

**Published:** 2024-11-19

**Authors:** Fatemeh Safizadeh, Marko Mandic, Ben Schöttker, Michael Hoffmeister, Hermann Brenner

**Affiliations:** 1 Division of Clinical Epidemiology and Aging Research, German Cancer Research Center (DKFZ), Heidelberg, Germany; 2 Medical Faculty Heidelberg, Heidelberg University, Heidelberg, Germany; 3 German Cancer Consortium (DKTK), German Cancer Research Center (DKFZ), Heidelberg, Germany

**Keywords:** Epidemiology, Epidemiology

## Abstract

**Background:**

General obesity commonly represented by body mass index (BMI) is an established risk factor for colorectal cancer (CRC). However, it is unclear to what extent this association is accounted for by central obesity. We aimed to evaluate the associations between BMI, waist-to-hip ratio (WHR), and waist circumference (WC) with CRC risk and to investigate if and to what extent these associations are independent from each other.

**Methods:**

Data from more than 500,000 male and female participants aged 40–69, recruited in the UK Biobank study between 2006 and 2010, were analyzed. Multivariable Cox proportional hazards models were fitted and hazard ratios (HR) and their 95% confidence intervals (CI) were calculated.

**Results:**

During a median follow-up of 12.5 years, of 460,784 participants, 5,977 developed CRC. Multivariable adjusted HRs (95% CIs) per standard deviation increase of BMI, WHR, and WC were 1.10 (1.07–1.13), 1.18 (1.14–1.22), and 1.14 (1.11–1.18), respectively. After mutual adjustment, the association with CRC was substantially attenuated for BMI (1.04 (1.01–1.07)), and remained substantially stronger for WHR (1.15 (1.11–1.20)). Furthermore, WHR showed strong, statistically significant associations with CRC risk within all BMI categories, whereas associations of BMI with CRC risk were weak and not statistically significant within WHR categories. BMI was also not associated with CRC risk in women and with rectal cancer after mutual adjustment. Conversely, WHR was strongly associated with CRC risk in both sexes and with both colon and rectal cancer risk before and after adjustment for BMI. BMI and WC could not be mutually adjusted for due to their high correlation.

**Conclusion:**

Central obesity is a much stronger predictor of CRC and may account for most of the CRC risk linked to obesity. Our findings also emphasize the need for incorporating measures such as WHR alongside BMI in clinical practice to improve obesity prevention and management.

## Introduction

Colorectal cancer (CRC) is the third most commonly diagnosed cancer worldwide, accounting for 10.4% of all cancer cases in men and 8.9% in women in 2022. It is also the second leading cause of cancer death in both sexes, contributing to 9.3% of total cancer deaths [1]. Overweight and obesity, commonly defined by higher body mass index (BMI) are established risk factors for CRC incidence [2, 3]. However, there seems to be substantial variation of risk by sex and CRC site, with associations being more pronounced among men and for colon compared to rectal cancer [2]. Moreover, of those systematic reviews and meta-analyses that stratified the results by sex and cancer site, the majority have found no association with rectal cancer in women [4–6].

BMI, as a measure of general obesity, has limitations such as inability to distinguish between muscle and fat mass and is insensitive to the fat mass distribution. Although much less assessed, there have been suggestions of stronger associations between central obesity measures, such as waist-to-hip ratio (WHR) or waist circumference (WC), and CRC risk [7, 8], and measures of central obesity have been suggested to better explain the underlying biological mechanisms related to cancer development [9]. However, the number of studies reporting on central obesity measures is much smaller compared to BMI, with partly inconsistent results when stratified by sex and cancer site. Furthermore, only a limited number of studies have evaluated the independent associations of BMI, WHR and WC with the risk of CRC, colon or rectal cancer [10–22]. Some of these studies were limited by small number of cases [10–13, 15], and the majority did not provide separate results for colon and rectal cancer or for both men and women. In addition, most of these studies adjusted models that included central obesity measures for BMI and not vice versa.

We aimed to investigate the association between various anthropometric measures, including BMI as the most commonly used general obesity measure, and WHR and WC as frequently used central obesity measures, with CRC risk in the large UK Biobank cohort. We paid particular attention to potential differences by sex and tumor site, as well as the independent associations of each anthropometric measure with CRC risk through mutual adjustment of these measures.

## Methods

### Study design and study population

The UK Biobank is a prospective population-based cohort study with more than 500,000 participants aged 40–69 years at recruitment between 2006 and 2010 [23, 24]. Details of the study protocol have been published elsewhere [23]. Briefly, comprehensive data collection included baseline questionnaires, physical measurements, sample assays, and longitudinal follow-up of various health outcomes via linkage to electronic health records, including cancer, death, and primary care data. The UK Biobank obtained ethical approval from the North West Centre for Research Ethics Committee (11/NW/0382) and collected signed informed consent from all participants. The current analysis was restricted to participants with no prior cancer diagnosis, except for non-melanoma skin cancer, and those with complete anthropometric data.

### Ascertainment of exposures

BMI was calculated as weight (kg) divided by height squared (m^2^) and WHR as WC (cm) divided by hip circumference (cm). At the initial assessment visit, weight and standing height were measured using a Tanita BC-418 MA body composition analyser and a Seca 202 height measure, respectively. Waist and hip circumferences were measured using a Wessex non-stretchable sprung tape measure [23].

BMI was categorized as <25 (normal), 25 to <30 (overweight), and ≥30 (obesity) according to WHO classification [25]. WHR and WC were classified using sex-specific categories [26] as follows: for WHR, men were categorized as <0.90 (normal), 0.90 to <0.95 (moderate), 0.95 to <1.00 (high), and ≥1.00 (very high), and women as <0.75 (normal), 0.75 to <0.80 (moderate), 0.80 to <0.85 (high), and ≥0.85 (very high). For WC (cm), men were classified as <88 (normal), 88 to <94 (moderate), 94 to <102 (high), and ≥102 (very high), and women as <72 (normal), 72 to <80 (moderate), 80 to <88 (high), and ≥88 (very high).

### Ascertainment of outcome

CRC cases in the UK Biobank were identified through linkage to national cancer registries and hospital data. Cancer incidence follow-up was available until 31 July 2019 for England, 31 December 2016 for Wales, and 31 October 2015 for Scotland from cancer registries. CRC cases diagnosed after these registry data censoring dates were ascertained using hospital episode statistics (HES), available until 30 September 2021 for England and 31 July 2021 for Scotland. Complete diagnosis data for Wales was available only until 31 March 2016, which was prior to the registry data censoring date. CRC diagnoses were coded according to the 10^th^ revision of the International Statistical Classification of Diseases (ICD-10), encompassing cancers of the colon (C18.0–18.9), rectosigmoid junction (C19), and rectum (C20).

### Statistical analysis

Baseline characteristics of the cohort were assessed using descriptive statistics. Spearman rank correlation coefficients were used to quantify correlations between the anthropometric measures, and contingency table analysis was employed to examine the joint distributions of categories of these measures. Hazard ratios (HR) and their 95% confidence intervals (CI) were calculated using Cox proportional hazard models to estimate the association between anthropometric measures and CRC risk. The date of the initial assessment visit was used as the entry time, with follow-up time defined by the time to first CRC diagnosis, death, loss to follow-up, or end of follow-up, whichever came first. The proportional hazards assumption was investigated by Schoenfeld residual plots, and no deviations were detected.

Three models with different levels of adjustment were fitted. Model 1 was adjusted for age at baseline (continuous, years) and sex (male, female). Models 2 and 3 were additionally adjusted for the following covariates: height (continuous, cm), ethnicity (white, other), Townsend deprivation index (continuous), educational qualifications (higher academic/professional, lower academic/vocational, none), smoking status (never, former, current), alcohol consumption (never, special occasions only, 1-3 times a month, once or twice a week, 3-4 times a week, daily or almost daily), physical activity determined by the International Physical Activity Questionnaire (IPAQ) [27] (low, moderate, high), fruit consumption (continuous, pieces/day), vegetable consumption (continuous, tablespoons/day), red and processed meat intake (less than once a week, once a week, ≥2 times a week), family history of CRC (no, yes), history of bowel cancer screening (no, yes), and regular use of non-steroidal anti-inflammatory drugs (NSAIDs) (no, yes).

Models 1 and 2 included only one of the anthropometric measures (BMI, WHR, or WC). Model 3 included both BMI and WHR to investigate their independent associations after mutual adjustment. Due to the high correlation between BMI and WC, the independent associations of these measures with the outcomes of interest could not be evaluated.

Subgroup analyses by age, sex, and smoking status were conducted. The associations of each anthropometric measure with the risk of colon cancer (C18.0-C18.9) and rectal cancer (C19 and C20) were also investigated separately and quantified using Cox models 2 and 3. Potential effect modifiers were evaluated by including interaction terms of BMI [continuous] or WHR [continuous] and each stratification variable. Differences between CRC sites were assessed using a test for heterogeneity (case-case analysis).

HRs (95% CIs) were calculated according to the classifications mentioned above, with the lowest category used as the reference group. Since the results from models 1 and 2 were very similar, HRs (95% CIs) obtained from model 1 were not displayed for subgroup and site-specific analyses. For comparability, HRs per one standard deviation (SD) increase in the anthropometric measures were also reported for all models.

In addition, a sensitivity analysis was conducted for the association between BMI and CRC risk, excluding participants with a BMI < 18.5 kg/m² (underweight), to ensure the robustness of the main results, as this group had been included in the reference category (individuals with normal BMI) due to their small number.

All analyses were conducted using SAS software version 9.4. Missing values for covariates (shown in Supplementary Table S1) were addressed using multiple imputation with PROC MI, and results from the five imputed datasets were combined using PROC MIANALYZE. P-values are two-sided and considered statistically significant if less than 0.05.

## Results

**Fig. 1:**
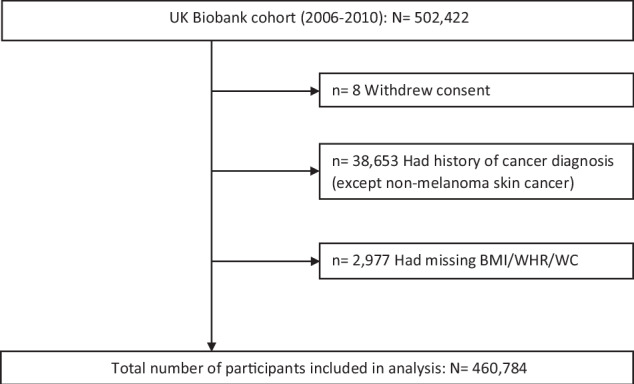
Flowchart of the study population. BMI body mass index, WC waist circumference, WHR waist-to-hip ratio.

The study population selection flowchart is shown in Fig. 1. Of 460,784 participants included in the analyses, 5977 developed CRC during a median follow-up of 12.5 years (interquartile range: 11.7–13.2). The study population had a mean (SD) age of 56.3 (8.1) years, comprised of 46.7% men and 53.3% women, and the majority (94.5%) of participants were white. Moreover, 67.1% had either overweight or obesity (BMI ≥ 25 kg/m^2^), and 49.4% and 60.5% had high or very high WHR (Men: ≥0.95, Women: ≥0.80) and WC (Men: ≥94 cm, Women: ≥80 cm), respectively. Baseline characteristics of the cohort are presented with descriptive statistics in Table 1 and Supplementary Table S2.

**Table 1:** Baseline characteristics of the cohort.

Characteristic	Total cohort (*n* = 460,784)	CRC cases (*n* = 5977)
Age at baseline (years)	56.3 ± 8.1	60.5 ± 6.7
Sex		
Male	215,325 (46.7)	3421 (57.2)
Female	245,459 (53.3)	2556 (42.8)
Height (cm)	168.6 ± 9.3	169.7 ± 9.2
Ethnicity		
White	433,251 (94.5)	5771 (97.1)
Other	25,316 (5.5)	174 (2.9)
Townsend deprivation index	−1.3 ± 3.1	−1.4 (3.1)
Educational qualifications		
Higher academic/professional	225,345 (49.5)	2679 (45.5)
Lower academic/vocational	152,643 (33.5)	1980 (33.6)
None	77,333 (17.0)	1233 (20.9)
BMI (kg/m^2^)	27.4 ± 4.8	28.0 ± 4.7
WHR	0.87 ± 0.1	0.90 ± 0.1
WC (cm)	90.4 ± 13.5	93.7 ± 13.6
Smoking status		
Never	252,835 (55.2)	2750 (46.2)
Former	157,159 (34.3)	2595 (43.6)
Current	48,485 (10.6)	608 (10.2)
Alcohol consumption		
Never	93,705 (20.4)	1566 (26.3)
Special occasions only	106,851 (23.2)	1370 (23.0)
1-3 times a month	118,908 (25.9)	1393 (23.4)
Once or twice a week	51,184 (11.1)	558 (9.4)
3-4 times a week	52,412 (11.4)	642 (10.8)
Daily or almost daily	36,695 (8.0)	434 (7.3)
Physical activity (IPAQ groups)		
Low	69,354 (18.7)	909 (19.0)
Moderate	150,744 (40.7)	1999 (41.8)
High	150,083 (40.5)	1879 (39.3)
Fruit intake (pieces/day)	3.1 ± 2.6	3.1 ± 2.9
Vegetable intake (tbsp./day)	4.9 ± 3.4	4.8 ± 3.0
Red meat intake		
Never	30,807 (6.8)	269 (4.6)
Less than once a week	155,220 (34.1)	2264 (38.3)
Once a week	98,699 (21.7)	1276 (21.6)
≥2 times a week	170,881 (37.5)	2104 (35.6)
Processed meat intake		
Never	42,469 (9.3)	397 (6.7)
Less than once a week	138,907 (30.3)	1695 (28.4)
Once a week	134,079 (29.2)	1789 (30.0)
≥2 times a week	143,624 (31.3)	2081 (34.9)
History of bowel cancer screening		
No	313,777 (69.4)	3756 (63.8)
Yes	138,479 (30.6)	2128 (36.2)
Family history of CRC		
No	401,626 (89.0)	4942 (84.3)
Yes	49,650 (11.0)	918 (15.7)
Regular use of NSAIDs/aspirin		
No	319,254 (69.3)	4091 (68.5)
Yes	141,514 (30.7)	1886 (31.6)

Data are expressed as mean ± SD or number of participants (percentage). Percentages might not add up to 100 percent due to rounding.

*BMI* body mass index, *CRC* colorectal cancer, *IPAQ* international physical activity questionnaire, *NSAIDs* nonsteroidal anti-inflammatory drugs, *tbsp* tablespoon, *SD* standard deviation.

Supplementary Table S3 shows the Spearman correlation coefficients between BMI, WHR, and WC. There were very high correlations between BMI and WC (0.80) and between WHR and WC (0.83). The contingency tables for the joint distribution of BMI and WHR (Supplementary Table S4) and BMI and WC (Supplementary Table S5) also demonstrated very strong concordance between BMI and WC categories. For example, 87.9% of participants classified as having obesity by BMI were also in the very high WC category, whereas only 0.1% of them were in the normal WC category.

Table 2 demonstrates the association between increased BMI, categorized as overweight and obesity compared to normal weight, with CRC risk, both with and without adjustment for WHR. The HR (95% CI) for overweight before adjustment for WHR was 1.13 (1.06–1.20), which attenuated to 1.05 (0.98–1.12) after adjustment, and became statistically insignificant. Obesity was strongly associated with CRC risk when BMI was not adjusted for WHR (HR 1.26, 95% CI: 1.17–1.35), but the association also became much weaker after adjustment (HR 1.11, 95% CI: 1.02–1.20). The association per SD increase in BMI followed a similar pattern, changing from 1.10 (1.07–1.13) without adjustment for WHR to 1.04 (1.01–1.07), a marginally statistically significant association, after adjustment. WHR and WC, classified as sex-specific categories, showed a more consistent and stronger association with CRC. Although there was a slight decrease in HRs (95% CIs) for all WHR categories when the model was additionally adjusted for BMI, the associations remained much stronger compared to those for BMI, with HRs (95% CIs) of 1.15 (1.11–1.20) and 1.04 (1.01–1.07) per SD increase in WHR and BMI, respectively. The very strong correlation between BMI and WC and the resulting multicollinearity prevented their mutual adjustment in CRC risk analyses. The results of the sensitivity analysis for the association between BMI and CRC across all models were nearly identical to those of the main analysis (Supplementary Table S6).

**Table 2:** Hazard ratios (HR) and their 95% confidence intervals (CI) for incident colorectal cancer risk associated with increased BMI, WHR, and WC.

Characteristic	*N* participants	*N* cases	HR (95% CI)		
			Model 1^a^	Model 2^a^	Model 3^b^
BMI^c^ (kg/m^2^)					
Normal	151,757	1602	Ref.	Ref.	Ref.
Overweight	196,316	2719	1.13 (1.06–1.20)	1.13 (1.06–1.20)	1.05 (0.98–1.12)
Obesity	112,711	1656	1.26 (1.18–1.35)	1.26 (1.17–1.35)	1.11 (1.02–1.20)
Per SD increase	460,784	5977	1.09 (1.07–1.12)	1.10 (1.07–1.13)	1.04 (1.01–1.07)
WHR^d^					
Normal	102,881	1011	Ref.	Ref.	Ref.
Moderate	130,266	1626	1.20 (1.11–1.30)	1.18 (1.09–1.28)	1.16 (1.07–1.26)
High	115,855	1603	1.29 (1.19–1.40)	1.27 (1.17–1.37)	1.23 (1.13–1.34)
Very high	111,782	1737	1.48 (1.36–1.60)	1.45 (1.34–1.57)	1.38 (1.26–1.51)
Per SD increase	460,784	5977	1.19 (1.15–1.23)	1.18 (1.14–1.22)	1.15 (1.11–1.20)
WC^e^ (cm)					
Normal	74,669	703	Ref.	Ref.	----------
Moderate	107,653	1226	1.19 (1.09–1.31)	1.17 (1.07–1.28)	----------
High	124,159	1694	1.29 (1.18–1.41)	1.25 (1.15–1.37)	----------
Very high	154,303	2354	1.46 (1.35–1.59)	1.41 (1.29–1.54)	----------
Per SD increase	460,784	5977	1.15 (1.12–1.19)	1.14 (1.11–1.18)	----------

Model 1 is adjusted for age and sex and all other models are additionally adjusted for height, ethnicity, socio-economic deprivation, education, smoking status, alcohol consumption, physical activity, fruit, vegetable, red meat and processed meat intake, history of bowel cancer screening, family history of CRC, and regular use of NSAIDs.

*BMI* body mass index, *CI* confidence interval, *CRC* colorectal cancer, *HR* hazard ratio, *NSAIDs* nonsteroidal anti-inflammatory drugs, *SD* standard deviation, *WC* waist circumference, *WHR* waist-to-hip ratio.

^a^The model includes BMI or WHR or WC.

^b^The model includes BMI and WHR.

^c^BMI (kg/m^2^) was categorized as: normal weight (<25), overweight (25–<30) and obesity (≥30).

^d^The following categories were used for WHR: Men; 0.90–<0.95 (moderate), 0.95–<1.00 (high) and ≥1.00 (very high). Participants with WHR <0.90 (normal) were the reference group. Women; 0.75–<0.80 (moderate), 0.80–<0.85 (high), ≥0.85 (very high) and participants with WHR <0.75 (normal) were the reference group.

^e^The following categories were applied to WC (cm): Men; 88–<94 (moderate), 94–<102 (high) and ≥102 (very high). Participants with WC <88 (normal) were the reference group. Women; 72–<80 (moderate), 80–<88 (high), ≥88 (very high) and participants with WC <72 (normal) were the reference group.

Tables 3 and 4 display the HRs (95% CIs) for CRC risk according to BMI within subgroups of WHR and WHR within subgroups of BMI, respectively. Consistent with the results shown in Table 2, WHR showed strong, statistically significant associations with CRC risk within each category of BMI, whereas associations of BMI with CRC risk were weak and not statistically significant within each category of WHR.

**Table 3:** Hazard ratios^a^ and their 95% confidence intervals of CRC risk according to BMI within subgroups defined by WHR.

Characteristic	WHR^c^				
		Normal	Moderate	High	Very high
BMI^b^	Normal	1.00 (ref)	1.00 (ref)	1.00 (ref)	1.00 (ref)
	Overweight	1.06 (0.93–1.22)	1.03 (0.92–1.15)	1.06 (0.92–1.21)	1.11 (0.93–1.33)
	Obesity	1.12 (0.84–1.51)	1.04 (0.89–1.22)	1.17 (1.00–1.36)	1.10 (0.92–1.31)
	Per SD increase	1.09 (0.99–1.19)	1.03 (0.96–1.10)	1.05 (0.99–1.11)	1.03 (0.98–1.08)

*BMI* body mass index, *CRC* colorectal cancer, *NSAIDs* nonsteroidal anti-inflammatory drugs, *SD* standard deviation, *WHR* waist-to-hip ratio.

^a^Adjusted for age, sex, height, ethnicity, socio-economic deprivation, education, smoking status, alcohol consumption, physical activity, fruit, vegetable, red meat and processed meat intake, history of bowel cancer screening, family history of CRC, and regular use of NSAIDs.

^b^BMI (kg/m^2^) was categorized as: normal weight (<25), overweight (25–<30) and obesity (≥30).

^c^The following categories were used for WHR: Men; 0.90–<0.95 (moderate), 0.95–<1.00 (high) and ≥1.00 (very high). Participants with WHR <0.90 (normal) were the reference group. Women; 0.75–<0.80 (moderate), 0.80–<0.85 (high), ≥0.85 (very high) and participants with WHR <0.75 (normal) were the reference group.

**Table 4:** Hazard ratios^a^ and their 95% confidence intervals of CRC risk according to WHR within subgroups defined by BMI.

Characteristic	BMI^b^			
		Normal	Overweight	Obesity
WHR^c^	Normal	1.00 (ref)	1.00 (ref)	1.00 (ref)
	Moderate	1.15 (1.02–1.30)	1.18 (1.04–1.34)	1.08 (0.79–1.47)
	High	1.14 (0.99–1.32)	1.23 (1.09–1.40)	1.24 (0.92–1.66)
	Very high	1.20 (0.99–1.45)	1.43 (1.25–1.63)	1.37 (1.03–1.83)
	Per SD increase	1.12 (1.04–1.21)	1.18 (1.11–1.26)	1.13 (1.05–1.21)

*BMI* body mass index, *CRC* colorectal cancer, *NSAIDs* nonsteroidal anti-inflammatory drugs, *SD* standard deviation, *WHR* waist-to-hip ratio.

^a^Adjusted for age, sex, height, ethnicity, socio-economic deprivation, education, smoking status, alcohol consumption, physical activity, fruit, vegetable, red meat and processed meat intake, history of bowel cancer screening, family history of CRC, and regular use of NSAIDs.

^b^BMI (kg/m^2^) was categorized as: normal weight (<25), overweight (25–<30) and obesity (≥30).

^c^The following categories were used for WHR: Men; 0.90–< 0.95 (moderate), 0.95–< 1.00 (high) and ≥1.00 (very high). Participants with WHR <0.90 (normal) were the reference group. Women; 0.75–<0.80 (moderate), 0.80–<0.85 (high), ≥0.85 (very high) and participants with WHR <0.75 (normal) were the reference group.

**Fig. 2:**
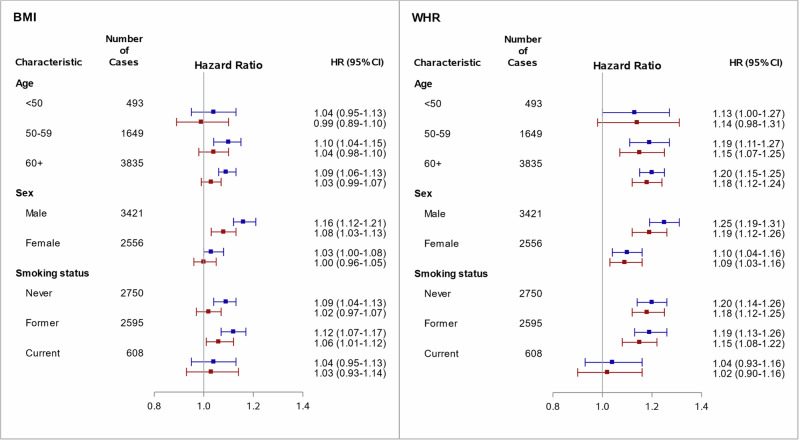
Forest plot of hazard ratios (HR) and their 95% confidence intervals (CI) for incident colorectal cancer risk per one standard deviation (SD) increase in BMI and WHR by age, sex and smoking status before (blue) and after (red) mutual adjustment. Models are adjusted for age, sex, height, ethnicity, socio-economic deprivation, education, smoking status, alcohol consumption, physical activity, fruit, vegetable, red meat and processed meat intake, history of bowel cancer screening, family history of CRC, and regular use of NSAIDs. BMI body mass index, CRC colorectal cancer, NSAIDs nonsteroidal anti-inflammatory drugs, SD standard deviation, WHR waist-to-hip ratio.

The results of the subgroup analyses are presented in Fig. 2. The association of BMI with CRC risk among men followed the same pattern as observed in the entire population, with WHR being a stronger predictor of CRC risk among men. Interestingly, no statistically significant association was found between BMI and CRC risk in women, either before (HR 1.03, 95% CI: 1.00–1.08) or after adjustment for WHR (HR 1.00, 95% CI: 0.96–1.04). Contrarily, WHR exhibited a robust and statistically significant association with CRC risk in women, even after adjusting for BMI. The associations between BMI and WHR with CRC risk among never and former smokers were comparable to those observed in the whole population, both before and after mutual adjustment. In the much smaller group of current smokers, weak and statistically insignificant associations were observed for both BMI and WHR in all models. Tests for interaction were statistically significant for sex for both BMI (*P*_interaction_ < 0.0001) and WHR (*P*_interaction_ < 0.0001), and for age for WHR (*P*_interaction_ = 0.0018).

**Fig. 3:**
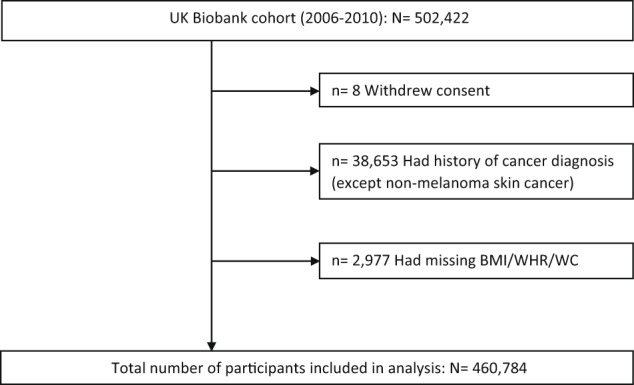
Forest plot of Hazard ratios (HR) and their 95% confidence intervals (CI) for incident colorectal cancer risk associated with increased BMI and WHR by cancer site, before (blue) and after (red) mutual adjustment. Models are adjusted for age, sex, height, ethnicity, socio-economic deprivation, education, smoking status, alcohol consumption, physical activity, fruit, vegetable, red meat and processed meat intake, history of bowel cancer screening, family history of CRC, and regular use of NSAIDs. BMI (kg/m^2^) was categorized as: normal weight (<25), overweight (25–<30) and obesity (≥30). The following categories were used for WHR: Men; 0.90–<0.95 (moderate), 0.95–<1.00 (high) and ≥1.00 (very high). Participants with WHR < 0.90 (normal) were the reference group. Women; 0.75–<0.80 (moderate), 0.80–<0.85 (high), ≥0.85 (very high) and participants with WHR < 0.75 (normal) were the reference group. BMI body mass index, CRC colorectal cancer, NSAIDs nonsteroidal anti-inflammatory drugs, SD standard deviation, WHR waist-to-hip ratio.

Figure 3 illustrates the HRs (95% CIs) for the association between anthropometric measures and the risk of colon and rectal cancer separately. Greater WHR was strongly associated with an increased risk for both colon and rectal cancer. The HRs were only slightly attenuated for colon cancer and remained almost unchanged for rectal cancer after adjustment for BMI. By contrast, the association between BMI and colon cancer risk was substantially attenuated, and the association between BMI and rectal cancer entirely disappeared after adjusting for WHR. The heterogeneity test for colon versus rectal cancer was statistically significant only for WHR (*P*_heterogeneity_ = 0.0003) and not for BMI (*P*_heterogeneity_ = 0.23).

## Discussion

In this study of more than 450,000 UK Biobank participants with a median follow-up of 12.5 years, BMI, WHR, and WC were associated with increased risk of CRC. After mutual adjustment, BMI’s association with CRC attenuated substantially, while remained robust for WHR. Furthermore, WHR showed strong, statistically significant associations with CRC risk within all BMI categories, whereas BMI’s associations were weak and not statistically significant within WHR categories. BMI was also not associated with CRC risk in women, either before or after adjustment for WHR, nor with rectal cancer after mutual adjustment. Conversely, WHR was strongly associated with CRC risk in both sexes, as well as with colon and rectal cancer risk, regardless of adjustment for BMI. Based on our findings, central obesity is associated with CRC, colon, and rectal cancer risk independent from general obesity and might account for most of the CRC risk associated with obesity.

The association between BMI, and to a lesser extent WHR and WC, with CRC, colon, and rectal cancer risk has been previously addressed [4–6, 28–32]. A 30% higher CRC risk for obesity compared to normal BMI [32], and 39% and 42% higher CRC risk for high WHR and WC, respectively, have been reported [8]. These outcomes are very similar to our results of 26%, 45%, and 41% increased CRC risk for obesity vs. normal BMI, and highest vs. lowest categories of WHR and WC, respectively. Moreover, our findings prior to mutual adjustment of anthropometric measures align with a recent UK Biobank study on various adiposity measures and 24 cancer types, including CRC [33].

Various pathophysiological mechanisms have been proposed to explain the relationship between excess adiposity and CRC [9]. These include older hypotheses such as insulin-insulin-like growth factor, inflammatory mediators and adipokines, and sex hormones, as well as more novel hypotheses like alterations in the colonic microbiota of individuals with obesity and systematic ectopic fat (central obesity). The latter suggests that visceral fat accumulation (i.e. greater WC) prompts considerable release of inflammatory markers and adipokines, contributing to metabolic dysfunction and cancer development. Since WHR and WC are more reflective of visceral fat than BMI, they pose higher metabolic disease risks even in individuals with normal weight, emphasizing their potentially superior relevance to CRC risk linked to obesity [9, 34]. Moreover, a UK Biobank study found that increased WHR and WC were associated with higher colorectal and colon cancer risk in men with normal BMI, highlighting the importance of central obesity in CRC risk compared to BMI [35].

Nevertheless, only a very limited number of studies have evaluated the independent association of different anthropometric measures with CRC risk. As most of these studies include a much smaller number of CRC cases, we will focus discussion on the very few larger ones.

In the European Prospective Investigation in to Cancer and Nutrition (EPIC) study, WHR was positively associated with colon and rectal cancer risk in both men and women before mutual adjustment [14]. When adjusted for BMI, the association with colon cancer remained relatively unchanged in women but was strongly attenuated and became statistically insignificant in men. Mutual adjustment was not reported for rectal cancer. These results are partly in line with our findings in women. However, in our study, the association was stronger and remained positive and significant in men after mutual adjustment. It is noteworthy that the EPIC study had a much shorter follow-up (average: 6.1 ± 1.7 years) and included much smaller numbers of cancer cases (colon: 984, rectal: 586) compared to our study (colon: 3997 rectal: 2006), which may partly explain the apparent heterogeneity in results.

In a study of 134,255 Chinese adults (72,972 women, mean age: 52.5 years, average follow-up: 11.0 years; 61,283 men, mean age: 55.4 years, average follow-up: 5.5 years), greater BMI, WHR, and WC were associated with higher risk of colon cancer in men only, with no associations found between any of the anthropometric measures and rectal cancer [36]. The risk associated with BMI was attenuated in analyses stratified for WHR, whereas the association between WHR and colon cancer remained positive and statistically significant in the high BMI stratum, which is in line with our findings. It is worth noting that overall means of BMI and WHR were much lower in this study compared to ours and other Western studies, with the majority of participants being in the normal weight range.

Finally, in a cohort of 28,191 women from Singapore, WHR showed a strong and positive association with CRC and colon cancer both before and after adjusting for BMI [21]. Consistent with our findings, the associations were stronger for WHR than for BMI. None of the anthropometric measures were significantly associated with rectal cancer. As with previous studies, the sample size and number of cases (colon: 404, rectal: 212) were much smaller than in our study.

Apart from its very large sample size, our study has several specific strengths. The comprehensive and detailed UK Biobank data enabled us to adjust thoroughly for known CRC risk factors. The large number of cases provided high levels of statistical power and precision, even in subgroup and site-specific analyses. Additionally, the anthropometric measures available in the UK Biobank were measured with highly standardized methods for all participants.

However, a number of limitations also deserve careful discussion. Firstly, only one-time measurements of anthropometric measures at baseline were considered. Neither previous lifetime history of overweight and obesity nor changes of anthropometric measures during follow-up, which are likely to affect CRC risk [37–39], could be considered. Furthermore, WHR and WC might not be the most accurate measures of central obesity, as WC includes both visceral and subcutaneous adipose tissue. Future research should more specifically address the role of each of these components. In addition, there is evidence of “healthy volunteer bias” in the UK Biobank cohort, with more health conscious and socioeconomically less deprived participants somewhat overrepresented. Absolute measures of prevalence and incidence of adverse health outcomes, including CRC incidence, may therefore underestimate prevalence and incidence in the general population to some degree. However, estimates of associations between risk factors and disease outcomes (such as the association between overweight and obesity and CRC incidence, which is the focus of our paper) are much less prone to such bias, and population representativeness of the study population is not a prerequisite for the validity of such associations. Moreover, despite comprehensive confounder adjustment, we cannot exclude the possibility of residual confounding due to imperfectly ascertained or unmeasured confounders and the lack of consideration of time-varying confounding. Finally, the predominantly white study population may limit the generalizability of findings to other ethnic backgrounds.

## Conclusion

Our investigation, the largest to date, independently assessed the associations of general and central obesity with CRC, colon, and rectal cancer risk. In a mainly European population, we found that central obesity likely contributes most to CRC risk associated with obesity. Previous estimates, primarily based on BMI studies, attribute about 11% of CRC cases to overweight and obesity in Western populations [40, 41]. However, our study reveals that WHR exhibits a much stronger association with CRC risk than BMI, suggesting that the CRC risk attributable to overweight and obesity may be substantially larger. These results also underline the importance of integrating additional anthropometric measures such as WHR alongside BMI into routine clinical practice for more effective prevention and management of obesity, whose prevalence is steadily increasing in many countries worldwide, in order to limit the global burden of CRC and many other obesity-related adverse health outcomes.

## Supplementary information

Supplemental material

## Data Availability

Data was re-used with the permission of the UK Biobank. This work used data provided by patients and collected by the NHS as part of their care and support. The UK Biobank is an open-access resource and bona fide researchers can apply to use the UK Biobank dataset by registering and applying at https://www.ukbiobank.ac.uk/enableyourresearch/apply-for-access. The data and analysis codes used for this study are going to be available on the UK Biobank website for registered researchers at the UK Biobank and an application fee.
