# Supplementary material for: Central obesity may account for most of the colorectal cancer risk linked to obesity: evidence from the UK Biobank prospective cohort
Source: Int J Obes (Lond). 2024 Nov 19;49(4):619–26. doi: 10.1038/s41366-024-01680-7 (PMC11999858; doi:10.1038/s41366-024-01680-7)
Supplement: Supplementary file 1 — Supplemental material [file 41366_2024_1680_MOESM1_ESM.docx]

**Supplemental material**

**Central obesity may account for most of the colorectal cancer risk linked to obesity: Evidence from the UK Biobank prospective cohort**

Fatemeh Safizadeh, Marko Mandic, Ben Schöttker, Michael Hoffmeister, Hermann Brenner

**Table S1.** Number of missing values for each variable in the total cohort.

**Table S2.** Baseline characteristics of the cohort.

**Table S3.** Spearman rank correlation coefficients between anthropometric measures.

**Table S4.** Joint distribution of study participants according to BMI and WHR.

**Table S5.** Joint distribution of study participants according to BMI and WC.

**Table S6.** Hazard ratios (HR) and their 95% confidence intervals (CI) for colorectal cancer risk associated with increased BMI after excluding participants with BMI<18.5 kg/m^2^.

**Table S1.** Number of missing values for each variable in the total cohort.

| **Variable** | **Number of missing values (%)** |
| --- | --- |
| Age at baseline | 0 (0.0) |
| Sex | 0 (0.0) |
| Height | 0 (0.0) |
| Ethnicity | 2,217 (0.5) |
| Townsend deprivation index | 566 (0.1) |
| Educational qualifications | 5,463 (1.2) |
| Smoking status | 2,305 (0.5) |
| Alcohol consumption | 1,029 (0.2) |
| Physical activity | 90,603 (19.7) |
| Fruit intake | 1,617 (0.4) |
| Vegetable intake | 3,794 (0.8) |
| Red meat intake | 5,177 (1.1) |
| Processed meat intake | 1,705 (0.4) |
| History of bowel cancer screening | 8,528 (1.9) |
| Family history of colorectal cancer | 9,508 (2.1) |
| Regular use of non-steroidal anti-inflammatory drugs (NSAIDs) | 16 (0.0) |

**Table S2.** Baseline characteristics of the cohort.

|  | **Total** | **Body Mass Index^1^ (kg/m^2^)** | | | **Waist-to-Hip Ratio^2^** | | | | **Waist circumference^3^ (cm)** | | | |
| --- | --- | --- | --- | --- | --- | --- | --- | --- | --- | --- | --- | --- |
|  |  | **Normal** | **Overweight** | **Obesity** | **Normal** | **Moderate** | **High** | **Very high** | **Normal** | **Moderate** | **High** | **Very high** |
|  |  | 32.9 | 42.6 | 24.5 | 22.3 | 28.3 | 25.1 | 24.3 | 16.2 | 23.4 | 27.0 | 33.5 |
| **Age at baseline (years)** | 56.3 (8.1) | 55.5 (8.2) | 56.8 (8.1) | 56.6 (7.9) | 54.2 (8.4) | 55.9 (8.1) | 57.1 (7.9) | 58.0 (7.6) | 54.4 (8.3) | 55.6 (8.2) | 56.8 (8.0) | 57.4 (7.8) |
| **Sex**  Male  Female | 46.7  53.3 | 35.7  64.3 | 54.2  45.8 | 48.6  51.4 | 60.4  39.6 | 51.5  48.5 | 43.5  56.6 | 32.0  68.0 | 56.0  44.1 | 42.3  57.7 | 50.4  49.6 | 42.4  57.6 |
| **Height (cm)** | 168.6 (9.3) | 167.9 (9.0) | 169.4 (9.4) | 168.0 (9.5) | 171.3 (9.0) | 169.5 (9.2) | 167.9 (9.2) | 165.9 (9.0) | 168.8 (9.0) | 167.9 (9.0) | 169.2 (9.4) | 168.5 (9.6) |
| **Ethnicity**  White  Other | 94.5  5.5 | 94.9  5.1 | 94.5  5.5 | 93.8  6.2 | 95.0  5.0 | 95.2  4.8 | 94.6  5.4 | 92.3  7.0 | 94.2  5.8 | 94.9  5.1 | 94.7  5.3 | 94.2  5.9 |
| **Townsend deprivation index** | -1.3 (3.1) | -1.5 (3.0) | -1.4 (3.0) | -0.8 (3.3) | -1.5 (3.0) | -1.5 (3.0) | -1.3 (3.1) | -0.8 (3.3) | -1.4 (3.1) | -1.6 (3.0) | -1.5 (3.0) | -1.0 (3.2) |
| Educational qualifications  Higher academic/professional  Lower academic/vocational  None | 49.5  33.5  17.0 | 57.3  30.2  12.6 | 48.3  34.3  17.5 | 41.1  36.8  22.1 | 58.8  30.8  10.4 | 52.0  33.6  14.4 | 46.6  34.7  18.7 | 41.0  34.7  24.3 | 58.2  30.2  11.6 | 54.1  32.4  13.5 | 48.8  34.1  17.1 | 42.5  35.5  22.0 |
| **Smoking status**  Never  Former  Current | 55.2  34.3  10.6 | 59.5  29.0  11.5 | 53.9  35.8  10.3 | 51.4  38.8  9.8 | 61.8  28.7  9.5 | 56.3  33.5  10.3 | 52.9  36.4  10.7 | 50.0  38.2  11.8 | 61.2  26.8  12.1 | 58.4  31.5  10.1 | 54.1  35.5  10.3 | 50.8  38.9  10.4 |
| **Alcohol consumption**  Never  Special occasions only  1-3 times a month  Once or twice a week  3-4 times a week  Daily or almost daily | 20.4  23.2  25.9  11.1  11.4  8.0 | 22.6  24.6  25.1  10.3  10.0  7.4 | 21.5  24.5  26.2  10.5  10.1  7.2 | 15.4  19.2  26.3  13.4  15.5  10.2 | 21.2  25.9  26.7  10.5  9.0  6.7 | 21.9  25.1  26.1  10.5  9.6  6.7 | 20.6  23.0  25.9  11.2  11.6  7.8 | 17.6  18.9  24.8  12.4  15.5  10.9 | 22.1  25.1  25.5  10.2  9.5  7.6 | 21.8  25.4  26.4  10.4  9.6  6.6 | 21.8  24.6  26.0  10.5  10.0  7.0 | 17.4  19.8  25.6  12.6  14.7  9.9 |
| **Physical activity (IPAQ groups)**  Low  Moderate  High | 18.7  40.7  40.5 | 14.6  40.9  44.6 | 17.9  41.0  41.2 | 26.2  40.1  33.7 | 13.9  38.6  47.5 | 17.1  40.7  42.2 | 20.2  41.7  38.2 | 24.2  41.9  34.0 | 12.8  37.3  49.9 | 14.8  40.9  44.3 | 18.0  41.6  40.4 | 25.3  41.6  33.1 |
| **Fruit intake (pieces/day)** | 3.1 (2.6) | 3.2 (2.7) | 3.0 (2.6) | 2.9 (2.5) | 3.2 (2.7) | 3.1 (2.6) | 3.0 (2.6) | 3.0 (2.6) | 3.2 (2.8) | 3.2 (2.6) | 3.0 (2.6) | 3.0 (2.5) |
| **Vegetable intake (tbsp./day)** | 4.9 (3.4) | 4.9 (3.3) | 4.9 (3.4) | 4.9 (3.4) | 4.8 (3.4) | 4.9 (3.4) | 4.9 (3.3) | 4.9 (3.3) | 4.8 (3.5) | 4.9 (3.4) | 4.9 (3.3) | 4.9 (3.4) |
| **Red meat intake**  Never  Less than once a week  Once a week  ≥2 times a week | 6.8  34.1  21.7  37.5 | 10.1  29.3  20.9  39.7 | 5.6  35.0  22.1  37.3 | 4.3  38.9  21.9  34.8 | 8.5  30.6  21.4  39.5 | 6.8  33.3  21.8  38.2 | 6.1  35.2  21.7  37.0 | 5.8  37.1  21.8  35.4 | 10.6  29.0  20.7  39.6 | 7.9  31.3  21.7  39.2 | 5.8  34.8  22.1  37.4 | 4.9  37.9  21.8  35.4 |
| **Processed meat intake**  Never  Less than once a week  Once a week  ≥2 times a week | 9.3  30.3  29.2  31.3 | 13.4  33.4  27.5  25.7 | 7.8  29.6  30.2  32.4 | 6.2  27.2  29.9  36.8 | 10.8  30.1  28.6  30.4 | 9.3  30.4  29.3  30.9 | 8.6  30.3  29.4  31.7 | 8.4  30.2  29.4  32.0 | 13.5  30.8  27.0  28.7 | 10.8  32.7  28.9  27.6 | 8.2  30.1  30.0  31.7 | 7.0  28.4  29.8  34.8 |

(continues)

(Table S1 continued)

|  | **Total** | **Body Mass Index (kg/m2)** | | | **Waist-to-Hip Ratio** | | | | **Waist circumference (cm)** | | | |
| --- | --- | --- | --- | --- | --- | --- | --- | --- | --- | --- | --- | --- |
|  |  | **Normal** | **Overweight** | **Obesity** | **Normal** | **Moderate** | **High** | **Very high** | **Normal** | **Moderate** | **High** | **Very high** |
| **History of bowel cancer screening**  No  Yes | 69.9  30.1 | 71.4  28.6 | 68.9  31.1 | 69.5  30.5 | 73.9  26.1 | 70.8  29.2 | 68.5  31.5 | 66.5  33.5 | 73.4  26.6 | 71.4  28.6 | 68.9  31.1 | 67.9  32.1 |
| **Family history of CRC**  No  Yes | 89.0  11.0 | 89.6  10.4 | 88.8  11.2 | 88.6  11.4 | 89.8  10.2 | 89.1  10.9 | 88.7  11.3 | 88.4  11.6 | 90.0  10.0 | 89.4  10.6 | 88.7  11.3 | 88.5  11.5 |
| **Regular use of NSAIDs/aspirin**  No  Yes | 69.3  30.7 | 76.0  24.0 | 69.3  30.7 | 60.2  39.8 | 75.6  24.4 | 71.3  28.7 | 67.6  32.4 | 62.8  37.2 | 77.3  22.7 | 73.9  26.1 | 69.5  30.5 | 62.0  38.0 |

Data are expressed as mean (SD) or percentage. Percentages might not add up to 100 percent due to rounding.

^1^BMI (kg/m^2^) was categorized as: normal weight (<25), overweight (25-<30) and obesity (≥30). ^2^The following categories were used for WHR: Men; 0.90-<0.95 (moderate), 0.95-<1.00 (high) and ≥1.00 (very high). Participants with WHR <0.90 (normal) were the reference group. Women; 0.75-<0.80 (moderate), 0.80-<0.85 (high), ≥0.85 (very high) and participants with WHR <0.75 (normal) were the reference group. ^3^ The following categories were applied to WC (cm): Men; 88-<94 (moderate), 94-<102 (high) and ≥102 (very high). Participants with WC <88 (normal) were the reference group. Women; 72-<80 (moderate), 80-<88 (high), ≥88 (very high) and participants with WC <72 (normal) were the reference group.

Abbreviations: BMI: body mass index; CRC: colorectal cancer; IPAQ: international physical activity questionnaire; NSAIDs: nonsteroidal anti-inflammatory drugs; tbsp: tablespoon.

**Table S3.** Spearman rank correlation coefficients between anthropometric measures among all participants, men, and women.

|  | **BMI** | **WC** | **WHR** |
| --- | --- | --- | --- |
| **All participants** |  |  |  |
| **BMI** | 1.00 |  |  |
| **WC** | 0.80 | 1.00 |  |
| **WHR** | 0.48 | 0.83 | 1.00 |
|  |  |  |  |
| **Men** |  |  |  |
| **BMI** | 1.00 |  |  |
| **WC** | 0.85 | 1.00 |  |
| **WHR** | 0.61 | 0.81 | 1.00 |
|  |  |  |  |
| **Women** |  |  |  |
| **BMI** | 1.00 |  |  |
| **WC** | 0.86 | 1.00 |  |
| **WHR** | 0.50 | 0.77 | 1.00 |

Abbreviations: BMI: Body mass index; WHR: Waist-to-hip ratio; WC: Waist circumference.

All *P*-values were <0.0001.

**Table S4.** Joint distribution of study participants according to BMI and WHR.

| **BMI^1^ category** | **N (col %)** | **WHR^2^ category** | | | |
| --- | --- | --- | --- | --- | --- |
|  |  | **Normal** | **Moderate** | **High** | **Very high** |
|  |  | N (row %) | N (row %) | N (row %) | N (row %) |
| **Normal** | 151,757 (32.9) | 62,157 (41.0) | 49,857 (32.9) | 26,608 (17.5) | 13,135 (8.7) |
| **Overweight** | 196,316 (42.6) | 35,791 (18.2) | 61,649 (31.4) | 55,730 (28.4) | 43,146 (22.0) |
| **Obesity** | 112,711 (24.5) | 4,933 (4.4) | 18,760 (16.6) | 33,517 (29.7) | 55,501 (49.2) |

^1^BMI (kg/m^2^) was categorized as: normal weight (<25), overweight (25-<30) and obesity (≥30). ^2^The following categories were used for WHR: Men; 0.90-<0.95 (moderate), 0.95-<1.00 (high) and ≥1.00 (very high). Participants with WHR <0.90 (normal) were the reference group. Women; 0.75-<0.80 (moderate), 0.80-<0.85 (high), ≥0.85 (very high) and participants with WHR <0.75 (normal) were the reference group.

Abbreviations: BMI: Body mass index; WHR: Waist-to-hip ratio.

**Table S5.** Joint distribution of study participants according to BMI and WC.

| **BMI^1^ category** | **N (col %)** | **WC^2^ category** | | | |
| --- | --- | --- | --- | --- | --- |
|  |  | **Normal** | **Moderate** | **High** | **Very high** |
|  |  | N (row %) | N (row %) | N (row %) | N (row %) |
| **Normal** | 151,757 (32.9) | 64,205 (42.3) | 61,403 (40.5) | 23,265 (15.3) | 2,884 (1.9) |
| **Overweight** | 196,316 (42.6) | 10,386 (5.3) | 45,248 (23.1) | 88,288 (45.0) | 52,394 (26.7) |
| **Obesity** | 112,711 (24.5) | 78 (0.1) | 1,002 (0.9) | 12,606 (11.2) | 99,025 (87.9) |

^1^BMI (kg/m^2^) was categorized as: normal weight (<25), overweight (25-<30) and obesity (≥30). ^2^The following categories were applied to WC (cm): Men; 88-<94 (moderate), 94-<102 (high) and ≥102 (very high). Participants with WC <88 (normal) were the reference group. Women; 72-<80 (moderate), 80-<88 (high), ≥88 (very high) and participants with WC <72 (normal) were the reference group.

Abbreviations: BMI: Body mass index; WC: Waist circumference.

**Table S6.** Hazard ratios (HR) and their 95% confidence intervals (CI) for colorectal cancer risk associated with increased BMI after excluding participants with BMI<18.5 kg/m^2^.

| **Characteristic** | **N participants** | **N cases** | **HR (95% CI)** | | |
| --- | --- | --- | --- | --- | --- |
|  |  |  | **Model 1^a^** | **Model 2^a^** | **Model 3^b^** |
| **BMI^1^ (kg/m^2^)** |  |  |  |  |  |
| **Normal** | 149,389 | 1,590 | Ref. | Ref. | Ref. |
| **Overweight** | 196,316 | 2,719 | 1.12 (1.06-1.20) | 1.11 (1.05-1.19) | 1.04 (0.97-1.11) |
| **Obesity** | 112,711 | 1,656 | 1.25 (1.17-1.34) | 1.24 (1.16-1.33) | 1.09 (1.00-1.18) |
| **Per SD increase** | 458,416 | 5,965 | 1.09 (1.06-1.12) | 1.09 (1.06-1.12) | 1.03 (1.01-1.07) |

Model 1 is adjusted for age and sex and all other models are additionally adjusted for height, ethnicity, socio-economic deprivation, education, smoking status, alcohol consumption, physical activity, fruit, vegetable, red meat and processed meat intake, history of bowel cancer screening, family history of CRC, and regular use of NSAIDs. ^a^ The model includes BMI. ^b^ The model includes BMI and WHR.

^1^BMI (kg/m^2^) was categorized as: normal (18.5-<25), overweight (25-<30) and obesity (≥30).

Abbreviations: BMI: Body mass index; CRC: Colorectal cancer; NSAIDs: nonsteroidal anti-inflammatory drugs; SD: Standard deviation.
